# When the Gates Swing Open Only: Arrhythmia Mutations That Target the Fast Inactivation Gate of Na_v_1.5

**DOI:** 10.3390/cells11233714

**Published:** 2022-11-22

**Authors:** Tamer M. Gamal El-Din

**Affiliations:** Department of Pharmacology, University of Washington, Seattle, WA 98195, USA; tmgamal@uw.edu

**Keywords:** arrhythmia, Na_v_1.5, inactivation gate, fast inactivation, IFM motif, late sodium current, LQT3, BrS1

## Abstract

Na_v_1.5 is the main voltage-gated sodium channel found in cardiac muscle, where it facilitates the fast influx of Na^+^ ions across the cell membrane, resulting in the fast depolarization phase—phase 0 of the cardiac action potential. As a result, it plays a major role in determining the amplitude and the upstroke velocity of the cardiac impulse. Quantitively, cardiac sodium channel activates in less than a millisecond to trigger the cardiac action potential and inactivates within 2–3 ms to facilitate repolarization and return to the resting state in preparation for firing the next action potential. Missense mutations in the gene that encodes Na_v_1.5 (SCN5A), change these time constants which leads to a wide spectrum of cardiac diseases ranging from long QT syndrome type 3 (LQT3) to sudden cardiac death. In this mini-review I will focus on the missense mutations in the inactivation gate of Na_v_1.5 that results in arrhythmia, attempting to correlate the location of the missense mutation to their specific phenotype.

## 1. Introduction

**Figure 1:**
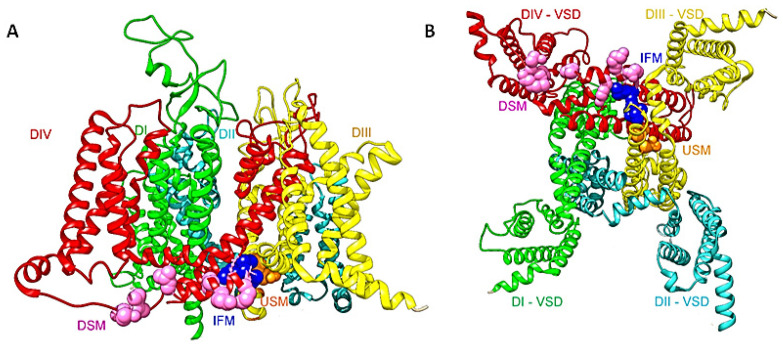
(**A**) A side view of the structure of the full length hNa_v_1.5 (PDB ID code 6LQA) [9]. DI is shown in green, DII in cyan, DIII in yellow, and DIV in red. The DIII-DIV linker IFM motif is shown as navy-blue spheres. Upstream (USM) and downstream (DSM) mutations are shown as orange and pink spheres respectively. (**B**) A bottom view shows the inactivation gate and the missense mutations that target this area. The same color code is used as in (**A**).

Voltage-gated sodium channels (Na_v_) are the main initiators and propagators of action potentials [1]. These proteins were discovered by Hodgkin and Huxley and have since been studied by a variety of biophysical and biochemical techniques. Eukaryotic Na_v_s are composed of a large pore-forming α subunit and a smaller auxiliary β subunits [2]. The α subunit contains 24 transmembrane segments organized in four homologous but not identical domains (DI-DIV) (Figure 1). The first four segments of each domain (S1–S4) comprise the voltage sensors modules (VSs), and the S5–S6 segments in addition to the pore loop between them (S5-P-S6) form the pore module (PM). The intracellular ends of the pore-lining S6 segments serve as the activation gate, which within 1 ms, opens the pore. The intracellular loop connecting domains III and IV (LIII-IV) forms the fast inactivation gate, which closes the pore in few milliseconds. Inactivation is triggered by the outward movement of the voltage sensors of DIII and DIV, which swing the IFM motif into a hydrophobic binding pocket [3,4,5,6] and as a result decouples the DIII S4–S5 linker from DIV S6. This efficient machinery of opening and inactivating the pore allows inward sodium current conduction within only 1–5 ms. As a result, mutation of any of the triple-hydrophobic motif in the DIII-IV, Ile-Phe-Met (IFM), or in their binding pocket impairs fast inactivation [7,8]. LQT3 and other cardiac arrhythmias are often caused by mutations that slow or prevent this efficient machinery of fast inactivation [9]. SCN5A mutations account for approximately 10% of cases of LQTS [10]. About 80% of the missense mutations in Na_v_1.5 led to either Long QT syndrome (LQT3) or Brugada syndrome (BRGDA1). More than 150 LQT3 mutations have been attributed to Na_v_1.5, most of them are in the III and IV pore domains and their intracellular linker [11]. Another group of these mutations are clustered around the fenestrations of neighboring domains [12,13]. The third group of arrhythmia mutations target the VSD of the channel where it causes gating pore current [14,15,16]. Persistent/late Na current, or gating pore current are the most common mechanisms behind the elongation of the QT interval that leads to LQT3.

Given the short length of the inactivation particle (IFM in DIII-DIV linker) and its binding pocket (formed by DIII S6 and DIV S5 and S6), it is striking that up to now, 13 arrhythmia mutations have been reported in this area. Most of these mutations cause LQT3 syndrome by impairing fast inactivation. This will lead to an increase in cardiac AP plateau duration, which will eventually cause early after-depolarizations (EAD) or delayed after-depolarization (DAD). Human and rat Na_v_1.5 structures showed the IFM motif bound to a pocket on the periphery of the activation gate. In rNa_v_1.5C, it was shown that the DIII S4–S5 linker shifts significantly toward the center of the activation gate, compared with the other three S4–S5 linkers. In this position, the activation gate cannot open wide enough for free conductance of hydrated Na^+^ [17]. Determining how missense mutations in the inactivation gate cause arrhythmia and how the location of the mutation affects the phenotype is the main theme of this review.

## 2. A Short History of the IFM Motif

The time scale of prokaryotic voltage-gated sodium channels inactivation is many folds slower than their eukaryotic counterparts [18,19,20,21]. These channels lack the fast inactivation machinery exist in eukaryotic sodium channels, but they retain the molecular mechanism for slow inactivation [19,22,23,24,25]. Fast inactivation was first described by Hodgkin and Huxley in the early fifties of the twentieth century [26]. Sodium channels quickly inactivate within 1–5 milliseconds after the channel opens. The duration of fast inactivation varies from one sodium channel isoform to another one to fulfill its local function. It is a critical step in the channel functional cycle that rapidly terminates each action potential to generate spikes of neuron firings in the excitable cells of eukaryotes. After Hodgken and Huxley proposed the existence of an inactivating “h” particle that inactivates the sodium channel, the first evidence was discovered by Armstrong and Bezanilla in the late seventies of the same century [27]. Further biochemical and biophysical studies confirmed that the intracellular loop connecting domains III and IV contains the fast inactivation gate [7,28] with the hydrophobic signature IFM (Isoleucine-Phenylalanine-Methionine) motif followed by a short helix. A “foot in the door” inactivation mechanism was suggested where the IFM motif physically occludes the open activation gate [27,29]. Recently, and after the success of resolving many eukaryotic sodium channels structures, a “door wedge” mechanism is hypothesized where the IFM motif binds to a hydrophobic receptor outside the activation gate formed by the S4–S5 linkers of domains III and IV, and the S6 of domains III/IV, which allosterically closes the pore by pushing the S6 towards the central axis of the pore [9]. This receptor site is only available when all the VSDs are activated because of the conformations of the S4–S5 linkers that are connected to the activation of S4 in the VSD. It has been reported that the movement of voltage sensing domain 4 is both necessary and sufficient for fast inactivation [4]. Since the VSDs activate sequentially in both voltage and time due to different voltage sensing thresholds and kinetics, VSD1 and VSD2 are the first to activate, while VSD4 is the last [5]. Fast inactivation is essentially triggered by the activation of VSD4 that couples the movement of S4 to the S4–S5 linker that forms the receptor site for the IFM motif to allosterically close the pore. The binding of the IFM motif squeezes S6 of domain III and IV by ~2–6 Å, respectively [3,17] to tilt inward toward the pore axis and close the activation gate.

## 3. The Inactivation Particle/Binding Pocket Mutations

**Figure 2:**
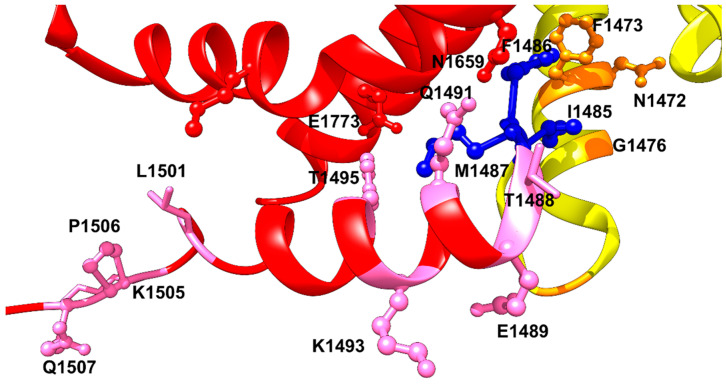
The IFM motif, shown in dark blue ball and sticks interacting with its binding pocket formed by S4–S5 linker, S5 and S6 of DIV, and the S6 of DIII. DIII is shown in yellow, and DIV in red. Upstream (USM) and downstream (DSM) mutations are shown as orange and pink ball and sticks, respectively.

Mutations in the inactivation gate pocket itself cause LQT3 (Figure 1 and Figure 2). A 2010 study showed a de novo deletion of phenylalanine 1486 (F1486del) of IFM motif. The infant patient harboring the deletion mutation had fatal long QT syndrome [30]. Biophysical characterization of F1486del showed that it reduces peak current density, impairs inactivation, and increases late current density [31]. If F1486 became mutated instead, such as in the case of mutation F1486L, it leads to an increase in late I_Na_ [32]. Likewise, mutation M1487L replaces methionine with a shorter side-chain amino acid which will lead to lowering the fitting of the IFM into its hydrophobic binding pocket (Figure 1 and Figure 2). In addition, it has been found that oxidation of methionine residue via reactive oxygen species (ROS), which may play a key role as a messenger in normal cell signal transduction and cell cycling, modulate sodium channel inactivation [33]. Thus, M1487L mutation may reduce the ability of ROS to tune sodium channel functionality.

## 4. Upstream Mutations (USM) Increase Channels Availability and Enhance Late Sodium Current

F1473 residue exists in S6-DIII; specifically twelve residues upstream, compared to IFM motif (Figure 1 and Figure 2). A de novo mutation F1473C was discovered in a newborn in 2007 where it caused a severe QT prolongation and differential responses to mexiletine, lidocaine, and flecainide [34]. The mutation caused a 6-fold increase in the amplitude of late sodium current compared to WT. It also increased the availability of the channels by shifting the steady-state inactivation curve by +10 mV and fastened the recovery from inactivation. A result which reflects improper docking of the IFM motif into its hydrophobic binding pocket. A similar mutation of F1473, but this time to S instead of C, also showed extreme QT prolongation with a significant increase in late sodium current and 20 mV rightward shift in the steady-state inactivation profile. It also showed incomplete inactivation and increased persistent sodium current by more than 20-fold compared to WT. It has been shown also that this mutation speeds the time course of recovery from inactivation. In addition, this mutation reduced the peak sodium current by more than 80% compared to WT [35].

On the other hand, there are four glycine residues at the N-terminal side of the IFM motif which play an important role for keeping the flexible movement of the inactivation particle. A mutation G1489K in the SCN1A gene (equivalent to G1476K in Na_v_1.5), which encodes for neuronal Na_v_1.1 channel, induces a two- to four-fold accelerated recovery from fast inactivation. G1481, five residues downstream from G1476, which is located at the tight turn of DIII S6 and the beginning DIII-DIV linker, is also crucial for maintaining the flexibility of N-terminus of the IFM motif (Figure 2). The mutation of G1481 to valine, with its bulky side chain results in two-fold increase of the late sodium current. It also shifts the steady-state inactivation rightward by 7 mV and speeds up the recovery from inactivation [36]. Another mutation, G1481E, has been shown to increase the current density and enhance the window current leading to LQT3 symptoms [10,37]. Most of the biophysical properties of the LQT3 mutations in the N-terminus side of the IFM motif converge to the same phenotype; increasing the availability of the channels by right-shifting the steady-state inactivation profile, increase in late sodium current, and faster recovery from inactivation (Table 1). All these biophysical features indicate an improper binding of the IFM motif to its hydrophobic receptor.

## 5. Downstream Mutations (DSM) Have a Mixed Phenotype of LQT3 and BrS; Increase in Persistent Sodium Current and Accelerated Rate of Fast Inactivation/Slow Inactivation

One residue from the IFM motif is T1488 (Figure 2). This residue forms a strong hydrogen bond with the backbone carbonyl of Q1318 in the S4–S5 linker of domain III. T1488R has been implicated in LQT3 mutations. Threonine to arginine change would disrupt the hydrogen bond with Q1318. A mutation in the next residue, E1489D, was reported as another LQT3 mutation [38]. The structure of hNa_v_1.5 shows that this residue, E1489, does not interact with any of the neighboring residues in the captured channel’s state [9]. However, it has been indicated that this residue is among the residues that exists in the calmodulin binding domain CaMBD, and thus one would expect that the short side chain of aspartate compared to glutamate would affect the affinity of calmodulin binding and thus affect Na_v_1.5 gating leading to arrhythmia [39]. However, the two mutations, T1488R and E1489D have not been studied functionally yet.

The Q1491H mutation is located at the C-terminal of the DIII-DIV linker, downstream from the IFM motif (Figure 2) (Table 1). It has been shown recently that this mutation shifts the V_1/2_ of activation by 9 mV rightward, with a significant difference in slope value [36]. It also changed the inactivation state of the channel by shifting the steady state inactivation curve by +20 mV. As a result, Q1491H mutation increases the window current, which can result in heart malfunctions such as LQT3. Consistent with the idea that abolishing or compromising fast inactivation would increase the slow inactivation entry rate, the Q1491H mutation facilitates entry into slow inactivation by 3-fold compared to WT, with a significant delay of recovery from the slow inactivated state. In addition, Q1491H mutation enhances the persistent current amplitude approximately 6-fold compared to WT. The recently published structure of hNa_v_1.5 revealed a strong hydrogen bond between Q1491 and N1659 in S5 segment of domain-III. The same interaction was previously resolved in the rat Na_v_1.5 structure which showed that Q1493, which is equivalent to Q1491 in hNa_v_1.5, forms a strong hydrogen bond with N1661 (N1659 in hNa_v_1.5). Since there is a strong hydrogen bond between Q1491 and N1659, Q1491H mutation may lead to a weaker interaction with N1659 because of a histidine shorter imidazole side chain. This weakened interaction will impair fast inactivation and lead to a gain-of-function effect. Likewise, K1493R mutation, which altered a highly conserved residue in the DIII-IV linker and was located two amino acids downstream from Q1491 demonstrated a significant positive shift in voltage-dependence of inactivation and a large ramp current near resting membrane potential, also indicating a gain-of-function effect like Q1491H mutation [44]. The hNa_v_1.5 structural model at the resting state showed K1493 wedged between the E1780 and E1784 on the end of S6 of DIV. Mutation K1493R may lead to bracing of these interactions, leading to stabilization of the resting state structure of the inactivation gate [3]. This explains why another CTD mutation, E1784K, may disrupt this interaction, resulting in accelerated rate of fast inactivation and a hyperpolarized shift of the inactivation curve, which indicates a faster entry into the slow inactivated state. The mutation also increased the late sodium current [45]. It has been shown that K1493E/E1784K double mutant rescued the channel from the late sodium current confirming that there is an electrostatic interaction between E1784 and K1493 [40]. However, in the activated state, K1493 side chain is pointing towards the cytosol without any clear interactions with the neighboring residues. K1493 is located in the C-terminal area that interacts with the EF-hand lobe of Calmodulin, indicating that a mutation of this residue will affect channel’s affinity towards Calmodulin. Residue Y1495 has been also demonstrated to play a crucial role in the binding of Ca^2+^/Calmodulin to cardiac sodium channel. Phosphorylation of Y1495 has been shown to abolish calmodulin binding and thus impairing the interaction with the inactivation gate receptor [46]. Despite that Y1495S LQT3 mutation preserves the ability to get phosphorylated but the change in the size of the side chain may result in weaker interaction of Ca^2+^/Calmodulin which may lead to elongation of the channel’s repolarization. On the other side, M1652 located in S4–S5 linker has been shown to interact with Y1495 and M1498 at the C-terminal side of the IFM motif. It has been shown that single point mutation M1652R enhances activation and causes a rightward shift in inactivation profile. In addition, it increases late sodium current of Na_v_1.5. Such a mutation would lead to a disruption of the hydrophobic interaction between these residues [41].

In the Cryo-EM structure of hNa_v_1.5, Y1495 interacts with the glutamate E1773 in S6 segment of DIV via forming a hydrogen bond with its hydroxyl group (Figure 2). An interaction that would be important in stabilizing the IFM motif in place, and it will be lost with a serine substitution at this position. L1501 and K1505 residues are predicted to interact with the CTD at the resting state. This was based on the Na_v_PaS-derived closed state model of Na_v_1.5 [9]. At the activated state, L1501 may interact with M1651 in S5 segment of DIV and help aligning alpha helix segment of the DIII-DIV linker to S5 and S6 of DIV to facilitate the positioning of the IFM motif into its binding pocket. K1505N mutation causes ~5 mV leftward shift in the steady-state inactivation curve, meaning that it reduces the availability of sodium channels. It has been also shown that K1505N mutation increases the persistent currents which was reduced by overexpression of CaM and FHF indicating that this residue is a site of interaction of CTD and the III-IV linker [42,43]. Another mutation in the downstream direction from IFM motif is the LQT3 triple mutation, ΔKPQ (1505–1507), which causes a three-residue in-frame deletion downstream of the inactivation gate and dramatically impairs fast inactivation [47]. This deletion mutation is in the flexible loop following the highly conserved α-helix in the DIII-DIV linker. One probable explanation of the structural basis of late sodium current caused by this deletion is that it would shorten the loop by approximately 13 A° and thus significantly limit the movement of IFM during cycles of activation and fast inactivation in the repeated cardiac action potentials [3,17] (Figure 2). As a result, these triple mutations lead to increase the window current significantly and a ~4 mV rightward shift in the steady-state inactivation profile.

Another LQT3 mutation which is in DIV S5 and the mutated residue forms a part of the IFM receptor site is I1660V. This mutation would change/reduce the perfect fit of the inactivation particle into its hydrophobic binding pocket. I1660V causes a reduced amplitude of the sodium peak current because the IFM motif seems to bind deeper, and as a result, makes the recovery from fast inactivation more difficult [48]. It is interesting to observe that most of the IFM downstream mutations have a mixed phenotype of LQT3 and BrS, which include an increase in persistent sodium current, accelerated rates of fast inactivation/slow inactivation, and a decrease in current density. It has been shown that E1784K mutation shifts the QV curve to a more hyperpolarized potentials indicating a faster voltage-dependent transitions for the S4 segment of DIV, the voltage sensor responsible for triggering fast inactivation [49]. One could speculate a similar mechanism for the other mutations downstream from the IFM motif, however more work is needed to reach a final conclusion about the effect of these mutations.

## 6. Conclusions

The impact of upstream mutations starting from F1473C/S to G1481E converge to the same biophysical phenotype; an increase in late sodium current, a rightward shift in steady-state inactivation (increase in channel availability), and faster recovery from inactivation (Table 1). All of these are gain-of-function effects. From a structural point of view, this indicates a failure of the IFM motif to dock perfectly with its hydrophobic binding site. This will lead to an increase in the net inward sodium, and eventually calcium currents, leading to early-afterdepolarization (EAD) or delayed-afterdepolarization (DAD) symptoms. On the other side of the IFM motif, mutations that target downstream residues tend to add more biophysical defects to the channelopathy, namely faster entry into slow inactivation and slower recovery from it. In addition, some of the downstream residues are involved in the interaction of the Na_v_1.5 C-terminus with Calmodulin, which would affect the inactivation kinetics of the channel. Most of these biophysical characteristics are loss-of-function that will eventually reduce the net inward sodium current, reducing action potential amplitude and velocity (Table 1). Some of these mutations have an overlapping phenotype with Brugada syndrome; something that is rare to find in upstream mutations. Categorizing the different mutations that cause arrhythmia is one step on the path to precision medicine, where medical interventions and/or medications have to be tailored to the patient’s genotype and not only their phenotype.

## Figures and Tables

**Table 1:** The most important Upstream (USM) and Downstream (DSM) muttions that target the inactivation gates of Na_v_1.5.

Location	Mutation	Biophysical Phenotype	Reference
**Upstream Mutations**	F1473C/S	○ Increases in late current. ○ Rightward shift in SSI. ○ Faster recovery from fast inactivation	[34] [35]
	G1481V	○ Increases in late current. ○ Rightward shift in SSI. ○ Faster recovery from fast inactivation	[36]
	G1481E	○ Increase in current density. ○ Leftward shift of GV curve. ○ Enhance the window current.	[10] [31]
**Inactivation particle IFM**	F1486del	○ Reduces peak current density. ○ Impairs inactivation. ○ Increases in late current.	[30] [31]
	F1486L	○ Increases late current.	[32]
	M1487L	○ Increases late current.	[33]
**Downstream** **Mutations**	E1489D	○ Affects Ca^2+^/Calmodulin binding.	[37] [38]
	Q1491H	○ Rightward shift in SSI. ○ Faster entry into slow inactivation. ○ Delayed recovery from slow inactivation.	[36]
	K1493R	○ Rightward shift in SSI. ○ large ramp current at resting membrane potential. ○ Slight increase in late sodium current.	[39]
	Y1495S	○ Affects Ca^2+^/Calmodulin binding.	[40]
	K1505N	○ Leftward shift in SSI. ○ Faster entry into slow inactivation. ○ Increase in late current. ○ Affects Ca^2+^/Calmodulin binding.	[41,42]
	ΔKPQ (1505–1507)	○ Rightward shift in SSI. ○ Increase window current	[43]

Upstream (USM), IFM motif, and downstream (DSM) mutations are shown as orange, blue, and pink colors, respectively.
